# A standardized Ashwagandha root extract alleviates stress, anxiety, and improves quality of life in healthy adults by modulating stress hormones: Results from a randomized, double-blind, placebo-controlled study

**DOI:** 10.1097/MD.0000000000035521

**Published:** 2023-10-13

**Authors:** Muhammed Majeed, Kalyanam Nagabhushanam, Lakshmi Mundkur

**Affiliations:** a Sami-Sabinsa Group Limited, Peenya Industrial Area, Bangalore, Karnataka, India; b Sabinsa Corporation, East Windsor, NJ, USA.

**Keywords:** anxiety, Ashwagandha root extract, CANTAB, cortisol, quality of life, serotonin, stress

## Abstract

**Background:**

The coronavirus disease-2019 (COVID-19) pandemic has resulted in a surge in stress, anxiety, and depression worldwide. Ashwagandha, an ayurvedic adaptogen has been traditionally used to manage stress, anxiety, and general well-being.

**Objective:**

We assessed the effect of Ashwagandha root extract (ARE-500 mg) standardized for 2.5% withanolides as per USP protocol with piperine (5 mg of 95% piperine) once daily for 60 days (12.5 mg withanolides/day) to alleviate stress and anxiety in healthy individuals with mild to moderate symptoms.

**Methods:**

A randomized, double-blind, placebo-controlled study was conducted for 60 days using ARE (n = 27) and placebo (n = 27) once daily at night at Narayana Institute of Cardiac Sciences, Bangalore, and Vijaya Super Specialty Hospital, Nellore, in India. The objectives of this study were to assess an improvement in perceived stress scale (PSS), generalized anxiety disorder (GAD-7), quality of life (QOL), cognitive scores in the Cambridge Neuropsychological Test Automated Battery (CANTAB), changes in salivary cortisol, urinary serotonin, dopamine, serum levels of nitric oxide (NO), glutathione (GSH) and malondialdehyde (MDA) from baseline to end of the study. Safety was evaluated by laboratory parameters, and by monitoring any incidence of adverse events.

**Results:**

54 individuals were randomized and 50 of them completed the study. The PSS, GAD-7, and QOL scores improved significantly in all the participants taking ARE compared to the placebo. The CANTAB analysis revealed a significant improvement in multitasking, concentration, and decision taking time in ARE compared to placebo. ARE was also associated with a greater reduction in the morning salivary cortisol and an increase in urinary serotonin compared to placebo. Serum levels of NO, GSH, and MDA were not significantly different. Biochemical and hematological parameters remained in the normal range in all participants and ARE was well tolerated during the study.

**Conclusion:**

The results of the study suggest that ARE with 2.5% withanolides can effectively improve stress and anxiety by reducing cortisol and increasing serotonin in healthy individuals with mild to moderate symptoms.

## 1. Introduction

The coronavirus disease-2019 (COVID-19) pandemic has affected almost every individual directly or indirectly, resulting in a substantial increase in stress, anxiety, and depression among people across the globe.^[1,2]^ Stress is a condition arising from physical and/or mental overload, while anxiety is a persistent excessive worry which continues even in the absence of external stimuli. Stress makes an individual feel nervous, anxious, tormented, and less capable of a normal response to environmental demands.^[3]^ A very fine line distinguishes stress from anxiety as both are emotional responses of an individual with overlapping symptoms. Prolonged exposure to stress can disturb the mental and physiological state of a person, leading to irreversible health issues like metabolic syndromes, cardiovascular issues, hypertension, endocrinological issues, anxiety, and visceral obesity.^[4,5]^

Adaptogens are herbal extracts that increase the ability of an organism to adapt to environmental stressors and decrease the damage from such factors. They are safe, non-habit forming, and balance the metabolic systems to potentiate the host response to external factors.^[6]^ *Ashwagandha*, or *Withania somnifera* Dunal, commonly known as Indian Ginseng or Winter Cherry holds a prominent position in the ayurvedic system of medicine and has been traditionally used as an adaptogen to promote vigor and vitality by enhancing muscle strength, endurance, and overall health.^[7]^ The major, pharmacologically important chemical constituents of the ashwagandha plant are the steroidal lactones and their glycosides, collectively known as withanolides.^[8–10]^ In animal stress models, ashwagandha has been shown to possess anxiolytic, antidepressant, and neuroprotective effects.^[11–13]^ Ashwagandha root extract (ARE) was reported to reduce stress in obese adults under chronic stress^[14]^ and anxiety and cortisol levels in chronically stressed adults.^[3,15]^

In a study using ashwagandha extract containing 5% withanolides, at a dose of 600 mg/day, serum cortisol was found to be reduced with improvement in stress and anxiety symptoms.^[16]^ In contrast, an extract from roots and leaves of Ashwagandha standardized for 35% withanolides showed no reduction in cortisol in healthy overweight men in a randomized study.^[17]^ In all these studies ashwagandha was well tolerated with minimal adverse effects. In the present study, we used a standardized extract of ashwagandha, prepared from dried roots, containing 2.5% withanolides (Shagandha), and analyzed by using a USP monograph (HPLC),^[18]^ and evaluated the efficacy and safety of this extract in improving the quality of life in healthy adults with stress and anxiety.

## 2. Materials and methods

### 2.1. Materials

**Figure 1.**
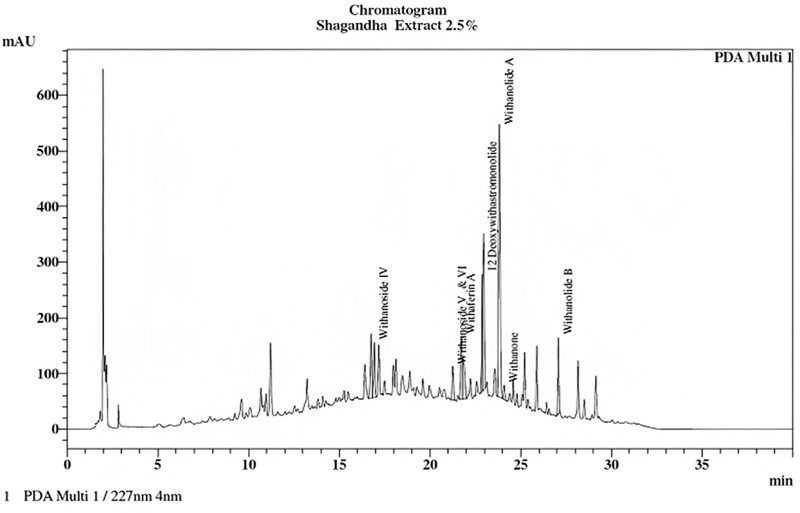
HPLC profile of Ashwagandha root extract analyzed as per USP method.

The test material was a root extract of *W somnifera* (Shagandha) standardized to contain 2.5% withanolides. A brief method of preparation of the extract is presented in the supplementary methods, http://links.lww.com/MD/K202. Each tablet contained 500 mg of extract and 5 mg of piperine (BioPerine), a safe food component known for increasing the bioavailability of actives.^[19,20]^ Microcrystalline cellulose capsules were used as a placebo. The product was provided by Sami-Sabinsa Group Limited. The HPLC profile of the extract is given in Figure 1.

### 2.2. Study design and ethics

A randomized, double-blind, placebo-controlled study was conducted from September 2020 to November 2021 at Narayana Institute of Cardiac Sciences, Bangalore, and Vijaya Super Specialty Hospital, Nellore, in India. The study was initiated only after receiving approval from the independent ethics committee of both the hospitals for Protocol No. CPL/83/ASH_STRS/I/AUG/19, V3.0,05-FEB-21. The study was conducted following Good Clinical Practice as required by the International Conference on Harmonization. The trial was registered prospectively with the Clinical Trial Registry of India (CTRI) with the registration number CTRI/2020/02/023321 on 13/02/2020.

### 2.3. Sample size

#### 2.3.1. Determination of sample size.

The sample size was calculated for an alfa error of 0.05 and a power of 80% based on the proportion of subjects with an effective response at the end of the treatment period. Based on the earlier study,^[3]^ and the deviation seen in the perceived stress scale (PSS), the sample size was calculated to be 25 subjects to be able to detect a difference of 10% to 15%. Considering 10% dropout, a sample size of 27 per arm was considered for the present study. A total of 54 subjects were enrolled and distributed in a 1:1 ratio into 2 arms, with 27 subjects in each arm.

### 2.4. Study population

#### 2.4.1. Inclusion criteria.

The study included adult participants in the age group of 21 to 54 years with a PSS score of <25 and more than 14, with anxiety as determined by the GAD-7 scores of <15 (mild-moderate stress and anxiety), with self-reported fatigue, insomnia, loss of appetite, and concentration. All the participants were free from any other psychological or psychiatric conditions or any chronic disease condition as assessed by the study physician and agreed to adhere to the assessments, visit schedules, and restrictions as described in this protocol.

#### 2.4.2. Exclusion criteria.

Participants with a history of multiple and/or severe allergies or anaphylactic reactions, alcohol abuse, excessive smoking; and hypersensitivity to the investigational product were excluded from the study. Other exclusion criteria were the presence of neurological or psychiatric disorders, chronic gastrointestinal and genitourinary diseases, severe immune deficiency, thyroid disorders, respiratory diseases, acute medical/surgical complications, and abnormal serum biochemical parameters. Participants who had used Ayurvedic/herbal/homeopathic/dietary supplements (including Vitamin E) or other alternative therapies in the last month, participated in any clinical study during the previous 3 months, and pregnant/lactating women were excluded from the study. Informed consent to participate in the study was signed by all the participants.

#### 2.4.3. Randomization and blinding.

Both the placebo and ARE capsules were identical in appearance to maintain the blindness of the study treatment. Subjects were randomized, using a predetermined randomization schedule generated using a computer-based randomization software (SAS 9.3), prepared by a statistician, independent of the sponsoring organization, and not involved in the conduct or reporting of the study. Block randomization was followed to generate an alphabetic code for the 2 groups to conceal allocations and to reduce any allocation bias. Both the investigational products were coded centrally with randomization codes. The participants were recruited by the principal investigator, who assigned them to the 2 groups as per the randomization sequence. The investigators, study staff, subjects, and statistician were blinded to the study. Sealed envelopes containing the randomization numbers were provided to the investigators, to be kept in a safe and access-controlled place to deal with any unforeseen emergency that warrants the breaking of blind codes. The codes were revealed to the statistician after data base lock.

#### 2.4.4. Intervention.

The study participants were instructed to consume ARE-500 mg, with 95% piperine-5 mg, (Total wt. 505 mg) or a comparable placebo containing microcrystalline cellulose (505 mg) per capsule once a day at bedtime for 60 days. Both the capsules were identical in appearance and weight.

The study period consisted of 4 scheduled visits, including a screening visit, a baseline visit (day 0), and 2 follow-up visits. The PSS and GAD-7 scores were assessed on screening visits as part of the inclusion criteria and on days 30 and 60. The general health condition of the participants, vital parameters, demographic details, and laboratory tests including hematological, biochemical, and urinary parameters, were evaluated on the screening day and days 30 and 60. The baseline visit was within 6 days of the screening for eligible candidates. During this visit and on day 60, the quality-of-life questionnaire, the Cambridge Neuropsychological Test Automated Battery (CANTAB) analysis, salivary cortisol, urinary dopamine, serotonin, and antioxidant markers were carried out. The schedule of enrollment, interventions, and assessments are provided in the Supplementary Table S1, http://links.lww.com/MD/K203

Additionally, a telephonic follow-up was conducted after 15 days from the date of the last visit to assess the occurrence of any adverse events (AEs) and overall well-being. Compliance was assessed by recording the number of capsules dispensed and consumed by the subject and those returned at each visit, in the case record form.

#### 2.4.5. Outcome.

The primary efficacy variables were changes in, the PSS, changes in the cognitive scores in the CANTAB Generalized anxiety disorder (GAD-7) scale, and quality of life from baseline/screening to final visit. The secondary endpoints also included changes in the salivary cortisol, urine serotonin, and dopamine, and serum levels of nitric oxide (NO), glutathione (GSH), and malondialdehyde (MDA). Safety was evaluated by laboratory parameters, and by monitoring any incidence of AEs.

### 2.5. Measures

#### 2.5.1. Stress and anxiety level.

The PSS Scale was used to assess the individual stress levels and the GAD-7 scale was used to evaluate the severity of generalized anxiety disorder. The PSS is a 14-item measure, widely used to assess the stress perception.^[19,20]^ The Generalized Anxiety Disorder Screener (GAD-7) is a 7-item self-report measure for anxiety symptoms. It has been used to monitor changes in symptoms over time and as a screening tool for anxiety,^[21,22]^ The stress and anxiety scale data were collected on the day of screening day 30, and day 60.

### 2.6. Quality of life

Quality of Life (QOL) was evaluated using the World Health Organization (WHO) QOL-BREF questionnaire, which is a shorter version of WHOQOL-100. The questions assess multiple statements about health and well-being.^[23]^ This questionnaire was assessed on baseline and day 60.

### 2.7. Cognitive functioning

CANTAB is a sensitive, precise, and objective measure of cognitive function. It includes tests of working memory, learning and executive function, visual, verbal, and episodic memory, attention, information processing and reaction time, social and emotion recognition, decision-making, and response control. The responses were recorded via touchscreen iPAD (MYLA2HN/A 8^th^ generation) and (MR7F2HN/A 6^th^ generation), controlled using CANTAB software (Cambridge Cognition, Cambridge, UK). All study participants were assessed individually. Since the study focus was anxiety and stress, the participants completed Motor Screening Task (MOT) to provide a baseline measure of the subject basic motor skills in terms of reaction times and accuracy, the Multitasking Test (MTT), and the Cambridge Gambling Task (CGT) to evaluate any changes in decision-making behavior (cantab.com.). The details of the tests are given in the supplementary methods section, http://links.lww.com/MD/K202.

### 2.8. Biomarkers and biochemical analysis

Fasting blood samples (approx.12.0 ml) were collected in collection tubes, serum was separated and stored at −70°C until use. Random urine samples (approx. 10 ml) were collected in sterile urine sample containers and analyzed within 24 hours. The participants were asked not to drink or eat anything for 60 minutes before saliva collection. Salivary samples were collected at 8.00 am and 4.00 pm in saliva collection tubes by the hospital nurse, samples were centrifuged, and the clear fluid was stored at −70°C until analysis. The participants stayed in the hospital during this time to complete the PSS, GAD-7, and QOL questionnaires and undertook the CANTAB analysis.

Nitric oxide, Glutathione, and Malondialdehyde in serum were assessed by colorimetric assay, salivary cortisol (8 am and 4 pm by ELISA) and urine Dopamine (Electrochemical method) and Serotonin (HPLC) levels were measured in Vijaya Diagnostic Center Private Limited and Anand Diagnostics, Bangalore. Regular serum biochemical tests and hematological and urine analyses were also carried out at the same labs.

### 2.9. Statistical analysis

Demographics and vital sign data were represented as means, standard deviation (SD), and percentages. The normality of the quantitative variables was analyzed using the Shapiro–Wilk test. The results were presented as median, range if the data was not normal. All the parameters were analyzed as changes from day 0 to day 60. The differences within and between the groups were compared by paired *T* test or Wilcoxon test, and unpaired or Mann–Whitney tests. Repeated measure 1-way analysis of variance (ANOVA) with Tukey Multiple Comparison Test was used for statistical analysis of the change in mean anxiety and stress scores at 3 time points within the group and 2-way repeated measure ANOVA with Bonferroni post-tests was used for between the groups comparison. All the statistical analysis was performed by STATA Software version 16.0 by an independent statistician blinded to the study groups. The level of significance was defined as 0.05.

## 3. Results

### 3.1. Demographic and baseline characteristics

**Figure 2.**
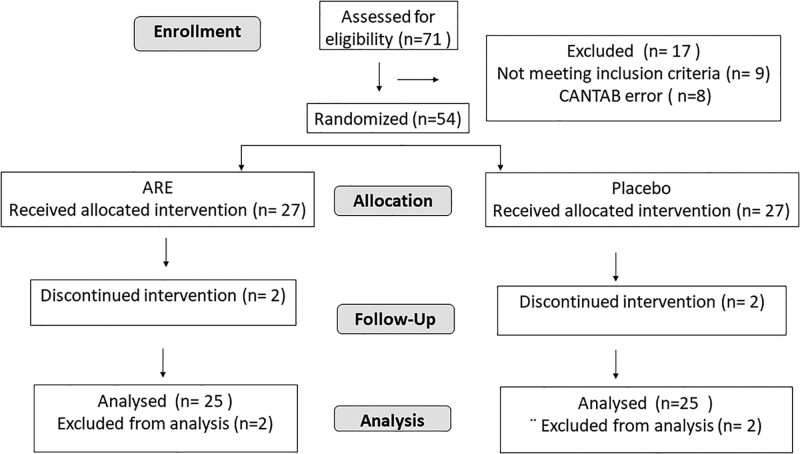
Consort diagram.

A total of 71 participants were screened and 62 were found eligible. For 8 participants, CANTAB data had errors and were disqualified, and 54 individuals were randomized for intervention, 27 each in ARE and placebo groups. Two participants from both groups discontinued the study due to personal reasons and 50 individuals (N = 25 in each group) completed the study and were taken for analysis (Fig. 2). The mean age of the subjects was 31.86 ± 9.20 years, 39 (78.0%) were men and 11 (22.0%) were women. Table 1 represents the baseline characteristics of the enrolled patients.

**Table 1:** Demographic and baseline characteristics of the study subjects.

Demographics	Placebo (n = 25)	ARE (n = 25)	Total patients (n = 50)	*P* value
Gender				.17
Male	17 (68.0%)	22 (88.0%)	39 (78.0%)	
Female	8 (32.0%)	3 (12.0%)	11 (78.0%)	
Age	31.76 ± 8.1	31.96 ± 10.3	31.86 ± 9.2	.94
Height (cm)	165.80 ± 7.6	168.52 ± 7.1	167.16 ± 7.4	.19
Weight (kg)	66.95 ± 9.4	67.11 ± 9.5	67.03 ± 9.4	.95
BMI (kg/m^2^)	24.20 ± 2.8	23.62 ± 3.1	23.91 ± 2.9	.49
Asian race	25 (100.0%)	25 (100.0%)	50 (100.0%)	-
Smoking history (yes)	0 (0.0%)	0 (0.0%)	0 (0.0%)	-
Alcohol history (yes)	0 (0.0%)	0 (0.0%)		-
Drug abuse history (yes)	0 (0.0%)	0 (0.0%)	0 (0.0%)	-
Systolic BP (mm Hg)	121.00 [120.0–122.0]	121.00 [120.0–121.0]	121.0 [120.0–122.0]	.66
Diastolic BP (mm Hg)	80.0 [80.0–81.5]	81.0 [75.0–81.5]	80.0 [80.0–81.25]	.98
Body temperature (°F)	97.97 ± 0.7	97.98 ± 0.6	97.98 ± 0.7	.97
Pulse rate (beats/min)	72.00 [70.0–77.0]	72.00 [71.0–74.0]	72.00 [70.7–74.2]	.61
Respiratory (rate/min)	20.36 ± 1.8	20.56 ± 2.2	20.46 ± 2.0	.73
Medical history-no	25 (100.0%)	25 (100.0%)	50 (100.0%)	-

Data is represented as Mean ± SD for anthropometric parameters, body temperature and respiratory rate. Gender, race, smoking, alcohol, and drug abuse history as number of participants N (%), while blood pressure and pulse rate are given as median and range. None of the participant had a history of smoking, alcohol, or drug abuse.

ARE = Ashwagandha root extract, BMI = body mass index.

### 3.2. Stress and anxiety assessments

**Figure 3.**
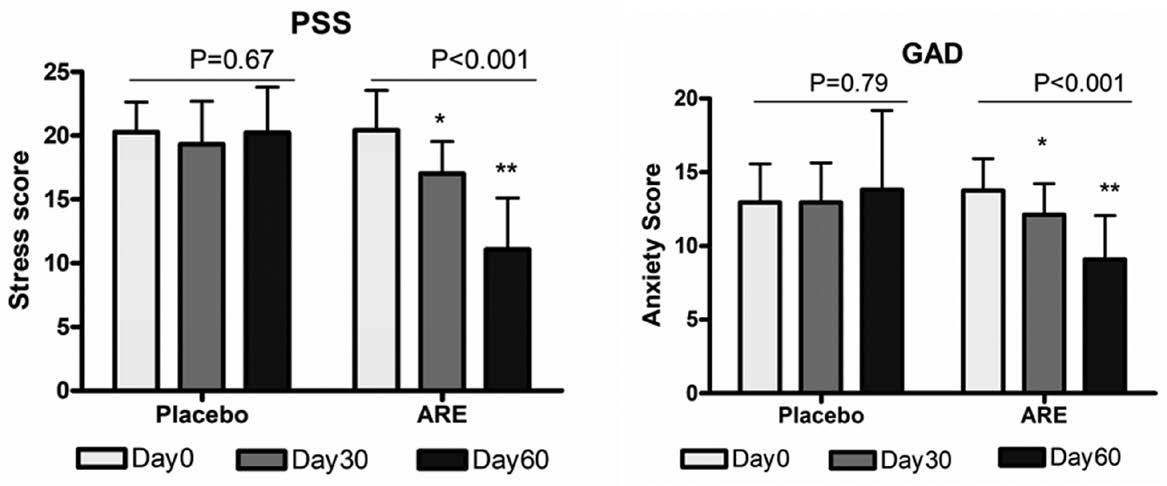
The mean GAD-7 and PSS scores at day 0 days 30 and 60 in the placebo and ARE groups. Repeated measures 1-way analysis of variance (ANOVA) with Tukey Multiple Comparison Test showed a significant difference from baseline to day 30 and day 60 in ARE. *P < .05 and **P < .01 for Tukey test between day 0 and days 30 and 60. ARE = Ashwagandha root extract, GAD-7 = generalized anxiety disorder.

GAD-7 and PSS scores were assessed at the baseline (day 0), visit 3 (day 30), and final visit (day 60) for all the participants. Figure 3 shows the mean scores at day 0, days 30, and 60 in the placebo and active groups. Repeated measures 1-way analysis of variance (ANOVA) showed a statistically significant (*P* < .001) reduction in stress (20.44 ± 3.12 to 11.08 ± 4.03) and anxiety (13.76 ± 2.17 to 9.09 ± 2.97) scores in the ARE group at day 60, while it was not significant in the placebo (20.28 ± 2.35 to 20.24 ± 3.57 for PSS and 12.96 ± 2.62 to 13.82 ± 5.37 for GAD-7). The difference between ARE and placebo was compared by repeated measures 2-way ANOVA with Bonferroni post hoc test at 3 different time points. The differences in stress scores between the groups were 2.57 (*P* < .05) on day 30 and 9.74 (*P* < .001) while the difference in anxiety scores was 1.30 and 5.12 (*P* < .001) on day 60 (Table 2).

**Table 2:** Stress and anxiety assessments.

Parameter	Time	Placebo	ARE	RMANOVA			Diff bet the groups at each time point	95% CI for the difference	*P* value
				SS	F	*P* value			
PSS	Day 0	20.28 ± 2.35	20.44 ± 3.12	639.0	34.94	<.001	−0.405	−2.57 to 1.76	>.05
	Day 30	19.32 ± 3.38	17.04 ± 2.52				2.57	0.41–4.74	<.05
	Day 60	20.24 ± 3.57	11.08 ± 4.03				9.74	7.57–11.91	<.001
GAD	Day 0	12.96 ± 2.62	13.76 ± 2.17	191.2	43.64	<.001	−0.45	−2.05 to 1.15	>.05
	Day 30	12.96 ± 2.68	12.12 ± 2.11				1.30	−0.29–2.89	>.05
	Day 60	13.82 ± 5.37	9.09 ± 2.97				5.13	3.53–6.73	<.001

Data is represented as Mean ± SD. The difference between the 2 treatment groups were compared using 2 -way repeated measure analysis of variance (RMANOVA) with Bonferroni post hoc test at 3 different time points. Time × treatment variation and degree of freedom = 2, for RM ANOVA is represented in the table.

ARE = Ashwagandha root extract, F = the ratio of the variance between the groups to the variance within the groups, GAD7 = generalized anxiety disorder, PSS = perceived stress score, SS = sums of squares.

### 3.3. Change in quality-of-life score from baseline to the final visit

The mean differences in physical, psychological, and social relationships, environmental, Q16, and the total QOL scores within the placebo and active groups are shown in Table 3. There were no significant mean differences in QOL scores within the placebo group compared to the baseline, while mean differences in QOL scores (except environmental health) were significant (*P* < .001) in the active group. A comparison of all QOL scores and total QOL scores between placebo and active groups reveals that mean score differences are statistically significant (*P* < .001 and *P* < .05) (Table 3).

**Table 3:** Mean differences in WHO-QOL scores.

Parameters	Group	Day 0 (mean ± SD)	Day 60 (mean ± SD)	Mean diff.	Group mean diff.	*P* value
Physical health score	Placebo	22.20 ± 2.08	21.84 ± 2.10	−0.36 ± 2.12	3.28 (1.67–5.03)	<.001
	Active	20.88 ± 1.39	23.80 ± 1.61***	2.92 ± 2.10		
Psychological health score	Placebo	18.36 ± 2.22	18.72 ± 1.86	0.36 ± 1.58	5.2 (3.82–6.52)	<.001
	Active	14.84 ± 2.30	20.40 ± 1.63***	5.56 ± 2.74		
Social relationships score	Placebo	9.16 ± 1.46	9.24 ± 1.01	0.08 ± 1.44	2.0 (1.19–2.80)	<.001
	Active	8.60 ± 1.08	10.68 ± 1.28***	2.08 ± 1.26		
Environmental health score	Placebo	24.68 ± 2.78	24.04 ± 2.82	(−0.64) ± 2.91	1.84 (0.59–3.99)	.016
	Active	24.84 ± 2.91	26.04 ± 1.93	1.20 ± 2.97		
Q16 health score	Placebo	3.28 ± 0.61	3.17 ± 0.84	(−0.08) ± 0.40	1.32 (0.85–1.56)	<.001
	Active	2.80 ± 0.61	4.05 ± 1.36***	1.24 ± 0.72		
Sum of QOL Scores	Placebo	74.40 ± 6.46	73.84 ± 5.60	(−0.56) ± 4.86	12.32 (10.29–16.04)	<.001
	Active	69.16 ± 4.24	80.92 ± 4.30***	11.76 ± 5.98		

Data is represented as Mean ± SD. WHOQOL: world health organization quality of life questionnaire.

*** Represents the significance (*P < .001) within the group from baseline to end of the study as determined by paired *T* test. The significance of the change in scores between ARE and placebo was computed by Mann–Whittney test and *P* values are represented in the table.

### 3.4. CANTAB

Cambridge Gambling Task, decision-making quality total (CGTMQMT, the mean latency from presentation of the task to the subject selection) score increased from 0.92 ± 0.11 to 0.96 ± 0.07 (*P* = .009) in ARE and 0.93 ± 0.13 to 0.96 ± 0.070 in placebo (*P* = .29). CGT mean decision time total merged (CGTDMMT), the time taken for taking a decision showed a decreasing trend in ARE group, while the latency time increased in the placebo. The risk-taking total merged (CGTRTKMT) scores were not statistically different from the baseline to the end of the study. In the delayed matching sample (DMS) tasks, the total number of times a subject chose the correct answer on their first box choice for trials where the target stimulus and response stimuli appeared on screen simultaneously (DMSTCS) and DMS Percent Correct (Simultaneous, DMSPCS) were used to understand the speed and correctness of the response. The percentage correct response increased from 86.92 ± 18.71 to 96.92 ± 7.36, *P* = .006 in ARE and from 93.08 ± 9.70 to 92.31 ± 9.92, *P* = .83 in the placebo, the change being significantly better (*P* = .02) in ARE group compared to placebo. The total correct responses were higher at the end of the study in ARE but were not significant. In multitasking, the total correct responses significantly increased, and incorrect responses significantly decreased in both ARE and placebo. The motor function was assessed by the mean latency for a subject to correctly respond to the on-screen stimulus during assessed trials (MOTML) decreased in ARE-supplemented participants while it increased in placebo, the difference in response being significantly better in ARE (*P* < .001) compared to placebo. In multitasking, the correct responses increased, and incorrect responses decreased in both ARE and placebo at the end of the study, compared to baseline (Table 4).

**Table 4:** CANTAB assessments.

Parameter	Placebo		ARE		Group mean diff. (95%CI)	*P* value
	Day 0	Day 60	Day 0	Day 60		
Cambridge Gambling Task (CGT)						
Mean decision time (CGTDMMT)	1372 ± 898.8	1686 ± 666.3	1793 ± 999	1721 ± 956.3	385.9 (−287.7 to 935.0)	.52
Change from day 0	314.1 ± 163.2		−71.80 ± 261.1			
Decision making Quality (CGTDMQMT)	0.93 ± 0.13	0.96 ± 0.07	0.92 ± 0.11	0.96 ± 0.07*	−0.016 (−0.038 to 0.013)	.27
Change from day 0	0.028 ± 0.024		0.045 ± 0.020			
Risk taking (CGTRTKMT)	0.58 ± 0.13	0.56 ± 0.11	0.55 ± 0.11	0.52 ± 0.07	0.016 (−0.03 to 0.066)	.54
Change from day 0	−0.02 ± 0.03		−0.033 ± 0.02			
Delayed Matching Sample (DMS)						
Percentage correct response (DMSPCS)	93.08 ± 9.70	92.31 ± 9.92	86.92 ± 18.71	96.92 ± 7.36*	−10.77 (−19.03 to 2.51)	.02
Change from day 0	−0.77 ± 2.59		10.0 ± 3.18			
Total correct response (DMSTCS)	4.65 ± 0.48	4.62 ± 0.49	4.42 ± 0.90	4.77 ± 0.51	0.38 (−0.053 to 0.82)	.31
Change from day 0	−0.038 ± 0.13		0.35 ± 0.17			
Motor Screening Task (MOT)						
Mean latency to respond (MOTML)	701.6 ± 295.4	799.6 ± 512.1	715 ± 272.4	663.5 ± 231.6	149.5 (−18.00 to 6.00)	.03
Change from day 0	98.07 ± 60.02		−51.47 ± 40.20			
Multitasking Test (MTT)						
Correct response (MTTTC)	133.7 ± 17.73	141.5 ± 16.37*	128.4 ± 17.44	143.1 ± 15.05*	−6.85 (−18.0 to 6.0)	.36
Change from day 0	7.84 ± 4.12		14.69 ± 3.91			
Incorrect response (MTTTIC)	22.81 ± 15.98	15.38 ± 14.25*	24.69 ± 15.19	15.15 ± 13.16*	2.11 (−6.00 to 12.00)	.72
Change from day 0	−7.42 ± 3.44		−9.54 ± 3.61			

Data is represented as Mean ± SD.

ARE = Ashwagandha root extract, CGTDMMT = Cambridge Gambling Task mean decision time total merged (The mean latency from presentation of the task to the subject selection), CGTMQMT = Cambridge Gambling Task, decision making quality total (the proportion of all trials where the subject chose the majority box color), CGTRTKMT = Cambridge Gambling task risk taking total merged (the mean proportion of current points gambled by the subject), DMSPCS = delayed, matching sample percentage correct (percentage of trials during which the subject chose the correct box on their first box choice), DMSTCS = delayed, matching sample total correct (total number of times a subject chose the correct answer on their first box choice), MMTTIC = multitasking total incorrect responses (number of trials for which the outcome was an incorrect response), MOTML = motor screening task mean latency (the mean latency for a subject to correctly respond to the stimulus on screen), MTTTC = multitasking total correct (number of trials for which the outcome was a correct response).

* Represents the significance (*P* < .05) within the group from baseline to end of the study as determined by paired *T* test. The significance of the change in scores from day 0 and day90 between ARE and placebo, by Mann–Whitney test are represented as *P* value in the table.

### 3.5. Biomarkers related to stress anxiety and oxidative stress

The salivary cortisol levels (morning and evening) showed a significant reduction in ARE group, while the levels were comparable in the placebo. The change in morning cortisol levels from baseline to end of the study was significantly different between ARE and placebo, while the change in evening cortisol levels did not show statistical significance (Table 5). The urinary serotonin levels increased in ARE but showed a decreasing trend in placebo. Urinary dopamine levels were observed to increase in both groups, but this increase was not significant. Changes in Glutathione and the marker of lipid peroxidation malondialdehyde did not significantly change from baseline to the end of the study in both groups.

**Table 5:** Biomarkers.

Parameters	Group	Day 0 (mean ± SD)	Day 60 (mean ± SD)	Mean diff.	Group mean diff, 95% CI	*P* value
Salivary Cortisol (Morning) nmol/L	Placebo	20.34 ± 5.92	21.46 ± 6.12	1.12 ± 0.93	3.69 (0.84–6.55)	.032
	Active	23.6 ± 4.86	21.02 ± 3.64*	−2.58 ± 1.05		
Salivary Cortisol (Evening) nmol/L	Placebo	2.28 ± 0.57	2.19 ± 0.64	−0.08 ± 0.08	0.36 (−0.14 to 0.87)	.31
	Active	2.63 ± 1.66	2.18 ± 0.77*	−0.44 ± 0.23		
Urinary Serotonin µg/g of creatinine	Placebo	49.10 ± 11.23	48.27 ± 22.15	−0.78 ± 3.78	−14.19 (−26.24 to −2.14)	.028
	Active	40.43 ± 13.26	53.83 ± 23.80*	13.40 ± 4.62		
Urinary Dopamine µg/ g of creatinine	Placebo	201.30 ± 65.58	227.10 ± 104.80	25.68 ± 14.16	14.97 (−20.30 to 50.24)	.89
	Active	217.90 ± 80.74	228.60 ± 93.00	10.71 ± 10.20		
Serum Glutathione µM	Placebo	2.55 ± 2.23	1.65 ± 1.81	−0.90 ± 0.26	0.81 (0.22–1.41)	.06
	Active	2.13 ± 1.7	2.04 ± 1.72	−0.09 ± 0.14		
Serum MDA nmol/ml	Placebo	2.88 ± 1.25	2.63 ± 1.23	0.24 ± 0.32	−0.21 (−0.98 to 0.55)	.24
	Active	3.21 ± 1.97	2.74 ± 1.25	0.47 ± 0.20		
Serum NO µM/L	Placebo	20.17 ± 13.8	20.47 ± 14.3	0.29 ± 0.49	0.34 (−1.60 to 2.27)	.34
	Active	23.11 ± 13.46	23.74 ± 13.12	0.62 ± 0.81		

Data is represented as Mean ± SD.

MDA = malonaldehyde.

* Represents the significance (*P* < .05) within the group from baseline to end of the study as determined by paired *T* test. The significance of the change in values from day 0 to day 60 between ARE and placebo were computed by Mann–Whittney test and the *P* values are represented in the table.

### 3.6. Safety

Mild discomforts were observed in 8 participants in ARE and 4 in placebo. All the events were transient and were resolved within 24 hours (Supplementary Table S2, http://links.lww.com/MD/K204). The study included hematology, fasting blood sugar, serum lipids, liver, and kidney function tests to assess the safety of the study product. The biochemical parameters were comparable between ARE and placebo except for LDL-cholesterol levels which showed a decrease in the ARE group (Supplementary Table S3–S5, http://links.lww.com/MD/K205, http://links.lww.com/MD/K206, http://links.lww.com/MD/K207).

## 4. Discussion

In this randomized double-blind, placebo-controlled study, consumption of 500 mg ARE (Shagandha) with 5 mg piperine for 60 days significantly improved the stress and anxiety symptoms in individuals with mild to moderate stress and anxiety, The Stress (PSS) and anxiety (GAD-7) scores improved in all the participants taking ARE while only 40% and 20% of the participants showed an improvement in stress and anxiety respectively in placebo. The quality of life of the participants improved significantly with ARE supplementation.

The anti-stress and anxiolytic activity of ashwagandha has been evaluated earlier in healthy participants, individuals with obesity-related stress, work stress, and stress associated with anxiety in different clinical studies.^[3,14,16,24]^ The dose of ashwagandha extract used in these studies ranged from 240 mg to 1000 mg, per day containing different percentages of withanolides. Our results are consistent with these studies, however, the dose used in our study was 500 mg of 2.5 % withanolides corresponding to 12.5 mg of withanolides compared to the dose of >30 mg effective withanolides used in some earlier studies.^[3,14,16,24]^ Most of these studies reported a significant decrease in stress by PSS scores and anxiety by depression anxiety, stress scale (DASS) scores. Our results corroborate these results at a lower dose. We used the GAD-7 scores to measure anxiety which is reported to be comparable to the DASS-21 to classify individuals as having above-threshold symptom severity.^[25]^

It is well-documented that anxiety affects cognitive performance,^[26]^ and acute stress impairs executive performance.^[27]^ Further, working memory plays a key role in the cognitive problems experienced by anxious people by limiting the resources necessary to perform goal-directed tasks.^[28,29]^ We used the CANTAB platform to assess the neurocognitive performance of participants in this study. A significant improvement in multitasking and concentration was observed with ARE supplementation compared to placebo. The time to take a decision was quicker in ARE group, while decision-making quality improved from baseline in participants supplemented with ARE, though it did not reach statistical significance compared to the placebo.

To understand the possible mechanism of ARE action, we evaluated salivary cortisol, as a stress marker. Salivary cortisol is known to be a valuable indicator of the hypothalamic-pituitary-adrenocortical axis activity, and its concentration was found to be directly proportional to the biologically active serum unbound cortisol concentration.^[30,31]^ Cortisol plays a central role in stress-induced HPA axis dysfunction and is elevated during stress. It follows a diurnal curve that peaks in the morning, 30 to 45 minutes after waking, and declines for the remainder of the day.^[32]^ The morning cortisol levels are reported to be positively associated with perceived stress in earlier studies.^[33,34]^ ARE consumption was associated with a reduction in morning cortisol in 64% of participants compared to 24% in placebo. The anti-stress activity of ashwagandha has been attributed to its effect on the glucocorticoid, cortisol in humans and corticosterone in rodents.^[35–37]^ Our results are consistent with earlier observations, albeit at a lower dose.

Serotonin (5-hydroxytryptamine, 5-HT) is a major neurotransmitter that plays a role in the maintenance of circadian rhythm, appetite, aggression, sensorimotor activity, mood, cognition, learning, and memory.^[38]^ Stress affects several aspects of serotonergic signaling in the brain and serotonergic drugs, in turn, can modulate the effects of stress.^[39]^ In correlation with the reduction in cortisol levels, there was an increase in serotonin levels in participants consuming ARE, suggesting an effect through the hypothalamic-pituitary-adrenal (HPA) axis. In preclinical studies, ashwagandha has been shown to influence serotonin activity,^[40,41]^ but clinical studies have not reported its effect on serotonin levels.

Dopamine is another central nervous system neurotransmitter that is involved in reducing depression. The association of dopamine with stress is complex as stress-induced elevations in cortisol levels have been directly correlated with amphetamine-induced dopamine release and administration of corticotrophin-releasing hormone was found to result in dopamine release.^[42,43]^ On the contrary, in a study assessing the effects of massage therapy, a decrease in cortisol was associated with an increase in serotonin and dopamine levels.^[44]^ We observed a nonsignificant increase in dopamine levels in both placebo and ARE, suggesting a minimal effect of ARE on dopaminergic pathways.

Stress-induced production of nitric oxide in the hippocampus can negatively alter adaptation to stress and can induce cellular toxicity by generating free radicals.^[45]^ Various physiological and physical stressors are known to induce the expression of nitric oxide synthetase in the brain.^[46]^ Serotonin is one of the neurotransmitters inhibiting its expression in the hippocampus.^[47]^ Surprisingly, we were unable to observe any changes in serum nitric oxide levels contrary to the preclinical study showing a reduction in nitric oxide-positive cells in rats treated with Ashwagandha.^[40]^ Glutathione levels and serum MDA levels also did not show any significant change in our study probably due to the small sample size and shorter study duration. We believe that a study for a longer duration could have shown some effect on antioxidant status.

Apart from these pathways, Ashwagandha supplementation was shown to reduce serum dehydroepiandrosterone sulfate in stressed adults.^[24]^ Other mechanisms include the anti-inflammatory effects of ashwagandha which may contribute to its anxiolytic and stress-relieving activity. The cumulative effect of different pathways is likely to contribute to the effects observed in the present study.

A few limitations of the study are worth mentioning. The study was carried out in healthy adults, with mild to moderate stress in southern India. Future studies in different ethnic populations with different degrees of anxiety and stress would be helpful. The present study examined the effect of ARE for 60 days in a relatively small population of 54 participants. Studies in a larger population and longer duration of supplementation and follow-up after termination will also help to understand the sustained effects of ARE in relieving stress and anxiety. The physiological response to stress and anxiety involved multiple, interdependent pathways. Future studies including the effect of ARE on the HPA axis, inflammation, and other hormones would delineate the mechanism of action of the supplement.

## 5. Conclusions

In conclusion, the result of this study suggests that ARE (Shagandha, standardized for 2.5% withanolides) at 500 mg with 5 mg of 95% piperine once a day could induce positive effects on stress and anxiety and improve the quality of life in healthy individuals with mild to moderate stress. A significant improvement could be observed in cognitive tasks, multitasking, and concentration in comparison to placebo. ARE significantly reduced cortisol levels and increased serotonin suggesting its mechanism of action through the HPA axis. The results of the study were comparable to earlier studies conducted with ashwagandha extracts containing a higher quantum of withanolides. Future studies in different ethnic populations and longer duration would substantiate our results.

## Acknowledgments

The authors thank Dr Vikneswaran G. from Narayana Institute of Cardiac Sciences and Dr S.V. Krishna Reddy from Vijaya Super Specialty Hospital, Nellore for conducting the study. The authors also thank Mr. Kamal Kammili, the statistician and all the clinical team associated with the study.

## Author contributions

**Conceptualization:** Muhammed Majeed, Kalyanam Nagabhushanam.

**Data curation:** Lakshmi Mundkur, Kalyanam Nagabhushanam.

**Formal analysis:** Lakshmi Mundkur.

**Funding acquisition:** Muhammed Majeed.

**Investigation:** Lakshmi Mundkur, Kalyanam Nagabhushanam.

**Methodology:** Lakshmi Mundkur, Kalyanam Nagabhushanam.

**Resources:** Muhammed Majeed.

**Supervision:** Kalyanam Nagabhushanam.

**Validation:** Lakshmi Mundkur, Kalyanam Nagabhushanam.

**Writing – original draft:** Lakshmi Mundkur.

**Writing – review & editing:** Lakshmi Mundkur, Muhammed Majeed, Kalyanam Nagabhushanam.

## Supplementary Material
