# Supplementary material for: A standardized Ashwagandha root extract alleviates stress, anxiety, and improves quality of life in healthy adults by modulating stress hormones: Results from a randomized, double-blind, placebo-controlled study
Source: Medicine (Baltimore). 2023 Oct 13;102(41):e35521. doi: 10.1097/MD.0000000000035521 (PMC10578737; doi:10.1097/MD.0000000000035521)
Supplement: Supplementary file 1 [file medi-102-e35521-s001.docx]

**Title:** **A Standardized** **Ashwagandha Root Extract Relieves Stress, Anxiety and Improves Quality of Life in Healthy Adults By Modulating Stress Hormones- Results From a Randomized, Double-Blind, Placebo-Controlled Study**

Muhammed Majeed^1,2^, Kalyanam Nagabhushanam^2^ Lakshmi Mundkur^1*^

^1^Sami-Sabinsa Group Limited, 19/1&19/2, I Main, II Phase, Peenya Industrial Area, Bangalore, Karnataka - 560 058 India.

^2^Sabinsa Corporation, 20 Lake Drive, East Windsor, NJ, 08520, USA

Supplementary Methods

**Preparation of Ashwagandha Extract**

Ashwagandha roots were pulverized and extracted with aqueous alcohol (20:80) at reflux conditions. The extract obtained was further concentrated to one fourth of its volume. This initial miscella was further processed to get an enriched Withanolides content. Calculated quantity of the excipient was added to obtain the desired 2.5% w/w of withanolides by HPLC

**Determination of sample size**

The sample size was calculated for an alfa error of 0.05 and power of 80% based on the proportion of subjects with an effective response at the end of the treatment period. Based on the earlier study [1], and the deviation seen in the Perceived Stress Scale and, , the sample size was calculated to be 25 subjects to be able to detect a difference of 10-15%. Considering 10% drop out, a sample size of 27 per arm was considered for the present study. A total of 54 subjects were enrolled and distributed in a 1:1 ratio into two arms, with 27 subjects in each arm.

**Details of CANTAB analysis**

**Delayed Matching to Sample:**

***Administration time***: 7 minutes

***Task format***: The participant is shown a complex visual pattern, that is both abstract and non-verbal (the sample), followed by four similar patterns, after a brief delay. The participant must select the pattern which exactly matches the sample. In some trials the sample and the choice patterns are shown simultaneously, in others there is a delay (of 0, 4 or 12 seconds) before the four choices appear.

*Outcome measures***:** Outcome measures include latency (the participant's speed of response), the number of correct patterns selected and a statistical measure giving the probability of an error after a correct or incorrect response.

**Cambridge Gambling Task**

***Administration time*:** Up to 18 minutes

***Task format*:** The participant is presented with a row of ten boxes across the top of the screen: some are red and some are blue. The ratio of red and blue boxes will vary between stages but there will always be one box that contains a yellow token. Participants must use the 'Red' and 'Blue' buttons at the bottom of the screen to choose the box colour in which they think the token is hidden.

In the assessed stages, participants start with 100 points and select a proportion of these points to bet on their decision. A circle in the center of the screen displays the current bet value, which will either incrementally increase or decrease (depending on the task variant selected). Participants press this button when it shows the proportion of their score they would like to bet. These points will either be added or taken away to their total score, depending on their decision and where the token is actually hidden.

***Outcome measures*:** Outcome measures include measurements of risk taking, quality of decision-making, decision time, risk adjustment, delay aversion and impulsivity.

**Multitasking Test**

***Administration time*:** 8 minutes

***Task format*:** The test displays an arrow which can appear on either side of the screen (right or left) and can point in either direction (to the right or to the left).

Each trial displays a cue at the top of the screen that indicates to the participant whether they have to select the right or left button according to the “side on which the arrow appeared” or the “direction in which the arrow was pointing”.

In some sections of the task this rule is consistent across trials (single task) while in others it may change from trial to trial in a randomized order (multitasking). Using both rules in a flexible manner places a higher demand on cognition than using a single rule.

Some trials display congruent stimuli (e.g. arrow on the right side pointing to the right) whereas other trials display incongruent stimuli, which require a higher cognitive demand (e.g. arrow on the right side of the screen pointing to the left).

***Outcome measures*:** Outcome measures for the Multitasking Test include response latencies and error scores that reflect the participant’s ability to manage multitasking and the interference of incongruent task-irrelevant information on task performance (i.e. a Stroop-like effect).

**Motor Screening Task**

***Administration time***: 2 minutes

***Task format*:** Colored crosses are presented in different locations on the screen, one at a time. The participant must select the cross on the screen as quickly and accurately as possible.

***Outcome measures*:** Outcome measures assess the participant's speed of response and the accuracy of pointing (selecting the cross).

**Results**

**Reference**

1. Chandrasekhar, K.; Kapoor, J.; Anishetty, S. A prospective, randomized double-blind, placebo-controlled study of safety and efficacy of a high-concentration full-spectrum extract of ashwagandha root in reducing stress and anxiety in adults. *Indian journal of psychological medicine* **2012**, *34*, 255-262, doi:10.4103/0253-7176.106022.
