# Supplementary material for: A standardized Ashwagandha root extract alleviates stress, anxiety, and improves quality of life in healthy adults by modulating stress hormones: Results from a randomized, double-blind, placebo-controlled study
Source: Medicine (Baltimore). 2023 Oct 13;102(41):e35521. doi: 10.1097/MD.0000000000035521 (PMC10578737; doi:10.1097/MD.0000000000035521)
Supplement: Supplementary file 2 [file medi-102-e35521-s002.docx]

**Table S1 STUDY EVENTS**

| Procedures | Screening visit  - 6 days | Baseline Visit  (Day 0) | Visit 3  (Day 30) | Visit 4  (Day 60) | Telephonic Follow-up  (After 15 days) |
| --- | --- | --- | --- | --- | --- |
| Informed Consent | X |  |  |  |  |
| Inclusion/Exclusion | X | X |  |  |  |
| Demographics* | X |  |  | X |  |
| Medical History | X |  |  |  |  |
| Physical Examination | X | X | X | X |  |
| Vitals | X | X | X | X |  |
| Concomitant Medications |  | X | X | X |  |
| PSS scale questionnaire | X |  | X | X |  |
| GAD-7 rating scale | X |  | X | X |  |
| Quality of life- WHOQOL |  | X |  | X |  |
| Anti-oxidative stress biomarkers  Nitric oxide, Glutathione, Malondialdehyde |  | X |  | X |  |
| Salivary Cortisol |  | X# |  | X# |  |
| CANTAB Analysis |  | X |  | X |  |
| Steer clear urine analysis for Dopamine & Serotonin. |  | X |  | X |  |
| Hematology, hepatic function, renal function test) | X |  | X | X |  |
| FBS | X |  |  | X |  |
| Thyroid functional Test | X |  |  |  |  |
| Lipid Profile | X |  |  | X |  |
| Urine analysis | X |  |  | X |  |
| Urine pregnancy test | X |  |  | X |  |
| IP dispensation |  | X | X |  |  |
| Subject diary dispensation |  | X |  |  |  |
| Collection of Used / Empty Bottles/Subject diary |  |  | X | X |  |
| Adverse Events |  |  | X | X | X |

*Age & height and level of education at screening only.

# To be conducted at 8 am and 4 pm.

Telephonic follow up will be done 15 days after the final visit.
