# Supplementary material for: A standardized Ashwagandha root extract alleviates stress, anxiety, and improves quality of life in healthy adults by modulating stress hormones: Results from a randomized, double-blind, placebo-controlled study
Source: Medicine (Baltimore). 2023 Oct 13;102(41):e35521. doi: 10.1097/MD.0000000000035521 (PMC10578737; doi:10.1097/MD.0000000000035521)
Supplement: Supplementary file 3 [file medi-102-e35521-s003.docx]

**Table S1:** **List of adverse events and their resolution.**

| Sl. No. | Screening Number | Subject Initial | Gender/AGE | AE NAME | Start Date | End Date | Outcome | Active/  Placebo |
| --- | --- | --- | --- | --- | --- | --- | --- | --- |
| 1 | 002003 | NS | F/36 | Nausea | 10-Feb-21 | 10-Feb-21 | Resolved | Active |
| 2 | 002007 | TBV | M/49 | Headache | 22-Mar-21 | 22-Mar-21 | Resolved | Placebo |
| 3 | 002009 | CHR | F/50 | Nausea | 01-Mar-21 | 01-Mar-21 | Resolved | Placebo |
| 4 | 002010 | KJN | M/31 | Nausea | 08-Apr-21 | 08-Apr-21 | Resolved | Active |
| 5 | 002014 | SGB | M/34 | Diarrhea | 27-Feb-21 | 27-Feb-21 | Resolved | Placebo |
| 6 | 002018 | LYA | M/32 | Nausea | 25-Mar-21 | 25-Mar-21 | Resolved | Active |
| 7 | 002021 | PSL | M/34 | Headache | 15-Mar-21 | 15-Mar-21 | Resolved | Placebo |
| 8 | 002025 | KTI | M/30 | Headache | 23-Feb-21 | 24-Feb-21 | Resolved | Active |
| 9 | 002029 | MSK | M/30 | Diarrhoea | 16-Apr-21 | 16-Apr-21 | Resolved | Active |
| 10 | 002032 | SA | M/25 | Diarrhea | 30-Mar-21 | 30-Mar-21 | Resolved | Active |
| 11 | 002033 | IAR | M/28 | Nausea | 22-Feb-21 | 22-Feb-21 | Resolved | Active |
| 12 | 002036 | RJA | F/50 | Nausea | 09-Apr-21 | 09-Apr-21 | Resolved | Active |

Description of mild adverse events experienced by participants during the study. M: Male, F: Female

**Table S1**

| **Parameter** | **Active** | **Placebo** |
| --- | --- | --- |
| Weight of capsule | 662.71 mg | 66 |
| Ashwagandha root extract 2.5% USP | 500 mg | - |
| Piperine | 5mg | - |
|  |  |  |
