# Supplementary material for: A standardized Ashwagandha root extract alleviates stress, anxiety, and improves quality of life in healthy adults by modulating stress hormones: Results from a randomized, double-blind, placebo-controlled study
Source: Medicine (Baltimore). 2023 Oct 13;102(41):e35521. doi: 10.1097/MD.0000000000035521 (PMC10578737; doi:10.1097/MD.0000000000035521)
Supplement: Supplementary file 4 [file medi-102-e35521-s004.docx]

**Table S2: Differences in the means of serum lipids and FBS levels within the placebo and active groups.**

| **Parameters** | **Placebo (n=23)**  (mean ± SD) | | **Active (n=24)**  (mean ± SD) | | **Group mean diff.** | **p-value** |
| --- | --- | --- | --- | --- | --- | --- |
|  | **Day 0** | **Day 60** | **Day 0** | **Day 60** |  |  |
| Total cholesterol, mg/dl | 152.30 ± 18.16 | 153.61 ± 22.93 | 151.13 ± 18.32 | 146.50 ± 16.55 | -5.93 | 0.090 |
| HDL-C, mg/dl | 43 ± 5.66 | 44.09 ± 5.12 | 41.88 ± 7.93 | 42.13 ± 7.53 | -0.84 | 0.492 |
| LDL-C, mg/dl | 83.17 ± 25.14 | 86.96 ± 28.80 | 86.79 ± 22.37 | 81.58 ± 22.28 | -8.99 | 0.041^*^ |
| VLDL-C, mg/dl | 22.22 ± 2.89 | 23.04 ± 6.89 | 23.25 ± 5.25 | 22.13 ± 4.46 | -1.95 | 0.239 |
| Triglycerides, mg/dl | 113.48 ± 11.46 | 115.83 ± 25.04 | 117.08 ± 26.49 | 120.17 ± 55.70 | 0.74 | 0.316 |
| FBS, mg/dl | 88.17 ± 5.60 | 91.17 ± 6.84 | 85.88 ± 5.18 | 88.79 ± 4.35 | -0.08 | 0.485 |

Data is represented as Mean± SD.HDL-C: High-density lipoprotein cholesterol, LDL-C: Low-density lipoprotein cholesterol, VLDL-C: Very low-density lipoprotein cholesterol, FBS: Fasting blood sugar.: *p<0.05
