# Supplementary material for: A standardized Ashwagandha root extract alleviates stress, anxiety, and improves quality of life in healthy adults by modulating stress hormones: Results from a randomized, double-blind, placebo-controlled study
Source: Medicine (Baltimore). 2023 Oct 13;102(41):e35521. doi: 10.1097/MD.0000000000035521 (PMC10578737; doi:10.1097/MD.0000000000035521)
Supplement: Supplementary file 5 [file medi-102-e35521-s005.docx]

**Table S3: Renal and hepatological parameters in the placebo and active groups.**

| **Parameters** | **Group** | **Day 0**  **(mean** ± **SD)** | **Day 60**  **(mean ± SD)** | **p-value** |
| --- | --- | --- | --- | --- |
| Total bilirubin, mg/dl | Placebo | 0.73 ± 0.13 | 0.74 ± 0.12 | 0.339 |
|  | Active | 0.75 ± 0.31 | 0.65 ± 0.24 | 0.106 |
| Alkaline phosphatase, IU/l | Placebo | 122.70 ± 28.05 | 128.05 ± 30.53 | 0.270 |
|  | Active | 122.79 ± 28.83 | 129.88 ± 35.78 | 0.227 |
| SGOT, U/l | Placebo | 23.70 ± 13.58 | 25.61 ± 13.08 | 0.314 |
|  | Active | 20.96 ± 5.01 | 22.79 ± 7.47 | 0.162 |
| SGPT, U/l | Placebo | 23.48 ± 10.80 | 25.35 ± 9.17 | 0.265 |
|  | Active | 23.04 ± 6.56 | 24.04 ± 6.64 | 0.30 |
| Urea, mg/dl | Placebo | 22.14 ± 3.76 | 21.37 ± 4.18 | 0.257 |
|  | Active | 21.0 ± 5.93 | 20.68 ± 4.05 | 0.414 |
| Uric acid, mg/dl | Placebo | 5.24 ± 0.78 | 5.21 ± 0.61 | 0.434 |
|  | Active | 5.09 ± 0.67 | 5.0 ± 0.90 | 0.355 |
| Serum creatinine, mg/dl | Placebo | 1.26 ± 1.02 | 1.04 ± 0.14 | 0.152 |
|  | Active | 1.02 ± 0.18 | 0.98 ± 0.18 | 0.269 |
| eGFR, ml/min/1.73m^2^ | Placebo | 88.22 ± 21.55 | 89.53 ± 19.48 | 0.415 |
|  | Active | 87.29 ± 19.76 | 89.93 ± 22.34 | 0.333 |
| Sodium, mmol/l | Placebo | 138.61 ± 2.59 | 136.96 ± 3.02 | 0.026 |
|  | Active | 139 ± 2.23 | 138.25 ± 2.29 | 0.128 |
| Potassium, mmol/l | Placebo | 4.90 ± 1.37 | 4.88 ± 1.72 | 0.483 |
|  | Active | 4.46 ± 0.35 | 4.66 ± 0.36 | 0.031 |
| Chloride, mmol/l | Placebo | 100.68 ± 2.07 | 100.04 ± 2.45 | 0.173 |
|  | Active | 101.22 ± 2.42 | 101.58 ± 3.15 | 0.326 |
| Specific gravity | Placebo | 1.02 ± 0.004 | 1.01 ± 0.004 | 0.051 |
|  | Active | 1.02 ± 0.01 | 1.02 ± 0.004 | 0.409 |
| pH | Placebo | 6.28 ± 0.45 | 6.30 ± 0.42 | 0.433 |
|  | Active | 6.33 ± 0.50 | 6.25 ± 0.53 | 0.290 |

Data is represented as Mean± SD: *p<0.05 and **p<0.01
