# Supplementary material for: A standardized Ashwagandha root extract alleviates stress, anxiety, and improves quality of life in healthy adults by modulating stress hormones: Results from a randomized, double-blind, placebo-controlled study
Source: Medicine (Baltimore). 2023 Oct 13;102(41):e35521. doi: 10.1097/MD.0000000000035521 (PMC10578737; doi:10.1097/MD.0000000000035521)
Supplement: Supplementary file 6 [file medi-102-e35521-s006.docx]

**Table S4: Hematological parameters in the placebo and active groups.**

| **Parameters** | **Group** | **Day 0**  **(mean** ± **SD)** | **Day 60**  **(mean ± SD)** | **p-value** |
| --- | --- | --- | --- | --- |
| Hemoglobin, g/dl | Placebo | 14.47 ± 0.91 | 14.68 ± 0.89 | 0.217 |
|  | Active | 14.16 ± 1.09 | 14.23 ± 1.01 | 0.408 |
| RBC, million/ul | Placebo | 4.89 ± 0.37 | 5.33 ± 1.65 | 0.107 |
|  | Active | 4.72 ± 0.37 | 4.79 ± 0.32 | 0.255 |
| Platelet count, lakhs/mm^3^ | Placebo | 2.61 ± 0.47 | 2.35 ± 0.59 | 0.056 |
|  | Active | 2.43 ± 0.57 | 2.53 ± 0.48 | 0.260 |
| Packed cell volume, % | Placebo | 44.10 ± 2.68 | 45.90 ± 2.60 | 0.013 |
|  | Active | 42.97 ± 3.23 | 44.24 ± 2.85 | 0.077 |
| Mean cell volume, fl | Placebo | 90.95 ± 3.57 | 92.31 ± 3.62 | 0.103 |
|  | Active | 91.23 ± 4.53 | 92.79 ± 4.75 | 0.125 |
| Mean platelet volume, fl | Placebo | 10.81 ± 0.65 | 11.27 ± 0.45 | 0.004 |
|  | Active | 10.78 ± 0.64 | 11.16 ± 0.52 | 0.015 |
| MCH, pg | Placebo | 27.62 ± 3.80 | 28.73 ± 0.76 | 0.089 |
|  | Active | 28.79 ± 1.93 | 28.95 ± 1.97 | 0.387 |
| MCHC, % | Placebo | 33.50 ± 1.34 | 33.93 ± 1.42 | 0.153 |
|  | Active | 33.68 ± 1.28 | 33.75 ± 1.42 | 0.420 |
| Total leukocyte count, cell/mm^3^ | Placebo | 7952.17 ± 1102.85 | 7699.57 ± 1014.55 | 0.212 |
|  | Active | 7924.58 ± 856.47 | 7746.25 ± 928.05 | 0.246 |
| Lymphocytes, % | Placebo | 32.34 ± 5.43 | 32.07 ± 1.28 | 0.407 |
|  | Active | 31.35 ± 5.40 | 33.79 ± 8.10 | 0.113 |
| Eosinophils, % | Placebo | 4.70 ± 6.70 | 2.80 ± 0.88 | 0.092 |
|  | Active | 3.23 ± 1.07 | 4.79 ± 5.84 | 0.103 |
| Monocytes, % | Placebo | 3.07 ± 3.46 | 3.71 ± 3.84 | 0.277 |
|  | Active | 3.07 ± 2.53 | 3.27 ± 2.71 | 0.396 |
| Neutrophils, % | Placebo | 62.46 ± 3.62 | 61.30 ± 4.11 | 0.158 |
|  | Active | 62.21 ± 6.47 | 60.55 ± 7.33 | 0.205 |
| Basophils, % | Placebo | 0.13 ± 0.30 | 0.13 ± 0.28 | 0.50 |
|  | Active | 0.096 ± 0.17 | 0.10 ± 0.18 | 0.43 |

Data is represented as Mean± SD: *p<0.05 and **p<0.01
